# Expression and Diagnostic Value of HE4 in Pancreatic Adenocarcinoma

**DOI:** 10.3390/ijms16022956

**Published:** 2015-01-29

**Authors:** Tianhe Huang, Shi-Wen Jiang, Liangyi Qin, Christopher Senkowski, Christian Lyle, Karen Terry, Steven Brower, Haibin Chen, Wayne Glasgow, Yongchang Wei, Jinping Li

**Affiliations:** 1 Department of Clinical Oncology, the First Affiliated Hospital, Xi’an Jiaotong University, Xi’an 710061, China; E-Mail: 15829550278@163.com; 2 Department of Biomedical Science, Mercer University School of Medicine, Savannah, GA 31404, USA; E-Mails: Jiang_S@mercer.edu or Jiangsh1@ memorialhealth.com (S.-W.J.); Glasgow_wc@mercer.edu or Glasgowwe1@ memorialhealth.com (W.G.); 3 Curtis and Elizabeth Anderson Cancer Institute, Department of Surgery, Memorial Health University Medical Center, Savannah, GA 31404, USA; E-Mails: senkoch1@memorialhealth.com (C.S.); terryka1@memorialhealth.com (K.T.); 4 Department of Clinical Laboratory, Shanghai YangSi Hospital, Shanghai 200135, China; E-Mail: qly12345@aliyun.com; 5 Department of Biological Sciences, Savannah State University, Savannah, GA 31404, USA; E-Mail: clyle0408@gmail.com; 6 Department of Surgery & Surgical Oncology, Mount Sinai Beth Israel Medical Center, New York, NY 10003, USA; E-Mail: sbrower@chpnet.org; 7 Department of Histology and Embryology, Shantou University Medical College, Shantou 515041, China; E-Mail: chenhb@stu.edu.cn; 8 Department of Obstetrics and Gynecology, Memorial Health University Medical Center, Savannah, GA 31404, USA

**Keywords:** HE4, pancreatic adenocarcinoma, biomarker, serum

## Abstract

Human epididymis protein 4 (HE4) is a recognized biomarker in ovarian and endometrial cancer and over-expressed in pancreatic adenocarcinoma. The diagnostic value of HE4 in pancreatic adenocarcinoma remains unknown. Here we elucidate mRNA, protein and serum level of HE4 in pancreatic adenocarcinoma. HE4 mRNA level in tumor adjacent tissues and pancreatic adenocarcinoma tissues were tested by real time-PCR. Tissue microarray containing normal, adenocarcinoma, and adjacent pancreatic tissue was tested by immunohistochemistry (IHC). Serum level of HE4, carbohydrate antigen 19-9 (CA19-9), carbohydrate antigen 15-3 (CA15-3) and carbohydrate antigen 125 (CA125) were detected by ELISA assay in control and tumor patients. Further we compared the sensitivity and specificity of determining HE4, CA19-9, CA15-3, and CA125 for diagnosis of pancreatic adenocarcinoma and assessed the complementary diagnostic value of HE4, CA19-9, CA15-3 and CA125. Real time PCR showed significantly increased HE4 mRNA level in pancreatic adenocarcinoma compared with control. Result of IHC showed that HE4 significantly higher expressed in the human pancreatic carcinoma tissues than in both normal and adjacent non-tumorous pancreatic tissues, and the staining intensity is inversely correlated with the clinical stage. HE4 was highly expressed in early stage of pancreatic adenocarcinoma. Serum HE4 level is higher in cases with pancreatic adenocarcinoma than in the controls. Serum HE4 levels could research to a sensitivity of 45.83% and specificity of 93.75% when the Cutoff was set at 4.59 ng/mL. The Combined HE4 and CA19-9 increased the sensitivity to 83.33%; and interestingly, the combination of HE4 with CA15-3 led to the most powerful sensitivity of 87.5%. Combined with CA19-9 and CA15-3, HE4 could be a potential biomarker to improve the diagnostic power for pancreatic adenocarcinoma.

## 1. Introduction

Pancreatic cancer accounts for approximately 3% of all cancers and 7% of cancer-related deaths. In the US alone, an estimated 46,420 people will be diagnosed with pancreatic cancer in 2014, and most patients will die of the malignancy with the current treatment. According to American Cancer Society, the one- and five-year survival rates after diagnosis are 25% and 5%, respectively, which makes pancreatic cancer one of the most dangerous malignancies. The most common type of pancreatic cancer is pancreatic adenocarcinoma [1]. Specific symptoms for pancreatic cancer such as jaundice and steatorrhea often do not appear until in an advanced stage. By the time of diagnosis, the cancer has usually spread to multiple organs. Thus, effective screening and early diagnosis is the key for improved management of pancreatic cancer patients.

Multiple serum markers, including carbohydrate antigen 19-9 (CA19-9), carbohydrate antigen 125 (CA125), and special AT-rich sequence-binding proteins 1 (SATB1) have been investigated [2,3,4]. It was reported that a panel composed of carcinoembryonic antigen (CEA), CA19-9, CA125 was useful for predicting outcomes of pancreatic cancer. CEA levels were correlated with tumor stages and high CEA levels may predict a poor overall survival [5,6]. Elevated serum CA19-9, CEA, CD44 variant 6 (CD44v6), and integrin-β1 levels were found to be closely related to the progression and metastasis of pancreatic cancers [7]. Gold, D.V., *et al.* have developed a murine monoclonal antibody, PAM4-based immunoassay to measure serum CA15-3 (mucin1) levels, and found that patients’ serum CA15-3 levels could be used to differentiate pancreatic cancer from pancreatitis [8]; Liu, X., *et al.* have examined the function of CA15-3 in pancreatic cancer cell lines, and suggested that CA15-3 may be involved in promoting pancreatic cancer cells proliferation by the interaction with p120 catenin [9]. However, up to now, none of these biomarkers has reached the sensitivity and specificity required for standard clinical practice.

Human epididymis protein 4 (HE4), a 25 kDa secreted glycoprotein, is specifically expressed in epididymis, lung and trachea tissues [10]. As a member of whey-acidic-protein (WAP) four-disulfide core domain (WFDC2) protein, HE4 carries protease inhibitor activity through interactions with serine proteases, Prss35 and Prss23, which are implicated in kidney fibrosis in a mouse model [11]. Numerous studies have demonstrated that HE4 is over-expressed in various malignant tissues including ovarian, endometrial, lung and gastric cancers [12,13]. The prognostic value of serum HE4 as a biomarker for ovarian and endometrial cancers has been well recognized [14,15]. The combination of serum HE4 with CA125 levels has shown an increased power for early detection of ovarian cancers [16]. Interestingly, Dr. Anastasi, E.’s group have found that serum HE4 levels could be used to monitor ovarian cancer patients’ outcome when it was combined together with carbohydrate antigen 72.4 (CA72.4) and CA125 biomarkers, benefiting the detection of ovarian cancer relapse during follow-up [17]; and serum HE4 levels, when combined with Multidetector Computed Tomographic (MDCT) imaging, could be applied to the follow up of the lymph node metastasis status for advanced ovarian cancer patients [18]. In a similar setting, a slight increase of serum CA125 combined with Magnetic Resonance Imaging (MRI) and Computed Tomographic (CT) imaging could be indicative for the progression of ovarian cancer [19]. HE4 serum concentrations significantly increased in endometrial cancer patients with metastases compared to early stage patients without metastases [20]. Wang *et al.* reported that serum HE4 could be a potential diagnostic marker for small cell lung cancers (SCLC) [21]. More interestingly, recent studies have indicated that HE4 may play an active role in the tumor pathogenesis and progression. Data from this and other groups suggested that HE4, when over-expressed in ovarian and endometrial cancer cells, is able to promote cell proliferation, adhesion, and invasion [22,23,24]. These new findings may partially explain why HE4 is highly expressed in many types of human malignancies.

Previous studies suggested that HE4 is overexpressed in pancreatic cancers. O’Neal *et al.* performed tissue microarray analysis and found that HE4 protein was detectable in 103 out of 220 (46.8%) pancreatic adenocarcinomas, whereas no HE4 expression was observed in pancreatic intraepithelial neoplasia [25], a precancerous lesion. They also analyzed HE4 levels and clinical outcomes, but found no evidence for the association of HE4 levels with patient survival. Using oligonucleotide microarray, Galgano *et al.* [26] demonstrated a moderate to high range of HE4 mRNA expression in pancreatic adenocarcinomas. In addition, their IHC results showed that five out of six pancreatic carcinoma tissues were HE4-positive. Faca *et al*. have showed that HE4 was increased in mouse pancreatic ductal adenocarcinoma (PDAC) plasma compared to normal plasma using proteomic analysis, and there was an increase of sera HE4 levels in 20 PDAC patients compared to 20 matched controls [27]. Recently, Brand *et al*. performed a bead-based immunoassay to measure HE4 serum levels, and set up the mean value of HE4 at 5.11, 2.07, 3.63 ng/mL for pancreatic cancer, benign pancreatic disease and healthy control individuals, respectively [28]. The goal of current study is to examine the relationship between HE4 and clinicopathological variables, and to assess the value of serum HE4 for the screening and/or diagnosis of pancreatic adenocarcinoma either alone or in combination with additional serum markers.

## 2. Result

### 2.1. Human Epididymis Protein 4 (HE4) mRNA Level Increased in Pancreatic Adenocarcinoma

**Figure 1:**
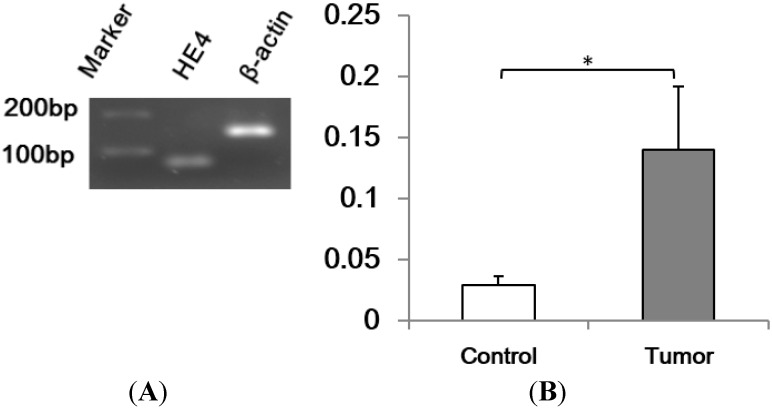
Human epididymis protein 4 (HE4) is over-expressed in pancreatic adenocarcinoma. Total RNA were extracted from pancreatic adenocarcinoma and adjacent tissues, and reverse transcription and real-time PCR were performed. (**A**) Real-time PCR final products were resolved in 2% agarose gel. The amplicon with single band pattern at predicted sizes (HE4, 68 bp; β-actin, 145 bp) indicates specific amplification of cDNA by real-time PCR. β-actin was used as an internal control; (**B**) The average HE4 mRNA levels were 4.8-fold higher in adenocarcinoma group (tumor) than the adjacent tissues group (Control) (* *p* < 0.05).

HE4 mRNA levels in cancer tissues (*n* = 14) were determined with real-time PCR following RNA extraction and reverse transcription. Pancreatic tissues with normal appearance collected from areas adjacent to malignant lesions were used as control (*n* = 32). Final real-time PCR products were resolved in agarose gel electrophoresis (Figure 1). The single band with predicted size indicated high specificity and accuracy of quantification with real-time PCR. Results showed a significantly higher HE4 mRNA levels of in cancer samples than adjacent tissues (*p* = 0.042).

### 2.2. Over-Expression of HE4 Protein in Pancreatic Adenocarcinoma Tissues

**Figure 2:**
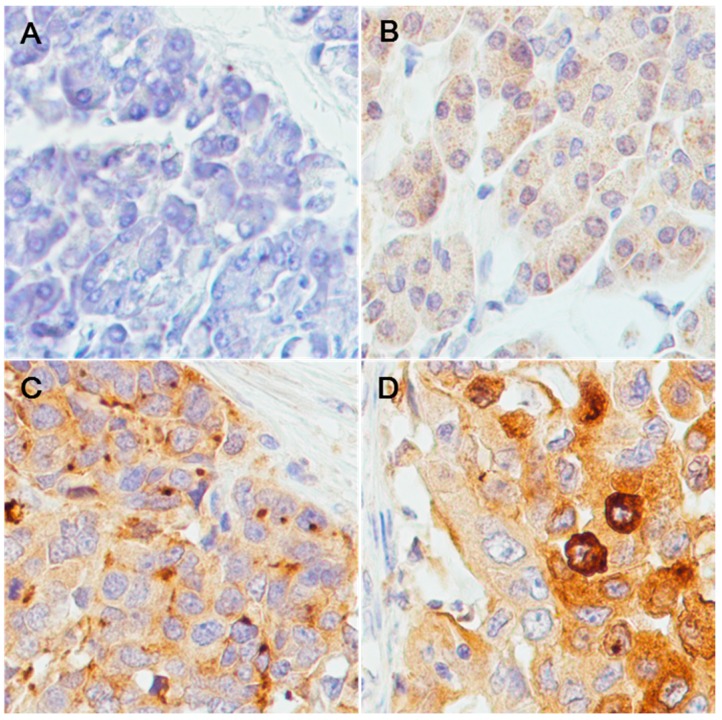
Representative results of HE4 immunohistochemisrty staining. Tissue cores were stained with antibodies against human HE4 and counterstained with hematoxylin. Negative, weak positive, and medium/strong positive staining was observed, respectively, in normal pancreatic tissues (**A**); adjacent non-tumorous tissues (**B**); and pancreatic adenocarcinoma tissues (**C**,**D**). Magnification power is at 400×.

To assess HE4 protein expression in pancreatic adenocarcinoma tissues, immunohistochemistry (IHC) was carried out in a pancreatic tissue microarray (TMA) containing normal pancreatic tissues, pancreatic adenocarcinoma tissue, and tumor adjacent tissues. After staining, the staining intensity was scored as −, +, ++, or +++ for each tissue core (Table 1). Figure 2 shows representative micrographs of the three types of tissues. Statistical analysis indicated a significant increase of HE4 expression in tumor tissues (*p* = 0.00) compared to normal or adjacent tissues, while very similar expression levels were found in normal or adjacent tissues. Correlation of the IHC score with the clinical stage and pathological grade was also examined. Interestingly, while no significant correlation was found between IHC score and cancer grade (*p* = 0.59), there was an inverse correlation between IHC score and clinical stage (Table 2), which suggested that HE4 expression may be associated with early stage of pancreatic adenocarcinoma.

**Table 1:** Immunohistochemistry score in pancreatic tissue microarrays. Each tissue microarray (TMA) core of 10 normal, 53 pancreatic tumor and 10 adjacent tissues was scored as negative (−), weak positive (+), medium positive (++), and highly positive (+++). Analysis on the HE4 level of three groups showed a significant increase of HE4 protein levels in tumor group compared to normal and adjacent group respectively. (*p* = 0.00, Kruskal–Wallis nonparametric correlations).

Diagnosis	−	+	++	+++	Total
Normal	4	6	0	0	10
Tumor	6	20	17	10	53
Adjacent	4	6	0	0	10
Total	14	32	17	10	73

**Table 2:** Correlation of immunohistochemistry (IHC) scores with clinical stages and pathological grades. A negative correlation was observed between HE4 IHC score and clinical stage (*p* = 0.02), but there was no correlation between HE4 IHC score and cancer grade (*p* = 0.59) in the same TMA core.

Level		−	+	++	+++	Total	Correlation
Stage	I	1	3	5	5	14	
	II	2	13	7	5	28	
	III	3	5	2	0	10	
	IV	0	0	2	0	2	
	total	6	21	16	10	53	*r* = −0.31, *p* = 0.02
Grade	1	2	1	2	0	5	
	2	0	10	9	3	22	
	3	4	9	6	7	26	
	total	6	20	17	10	53	*r* = 0.08, *p* = 0.59

### 2.3. Serum HE4 Is a Potential Biomarker for Pancreatic Adenocarcinoma Alone or in Combination with Classical Pancreatic Adenocarcinoma Biomarkers

**Figure 3:**
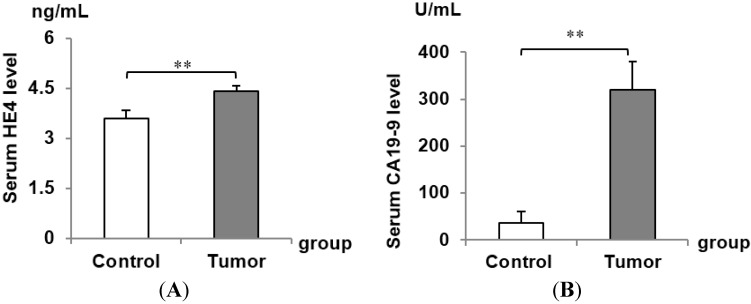

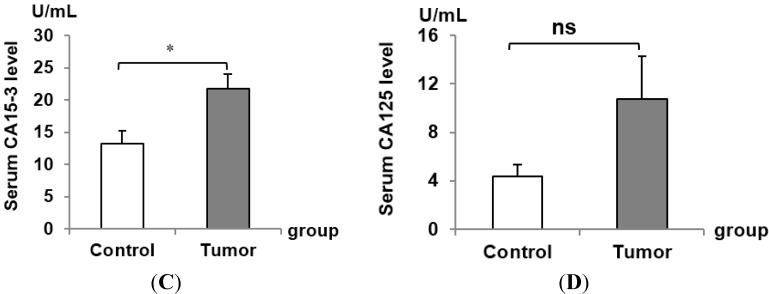
Serum level of HE4, carbohydrate antigen 19-9 (CA19-9), carbohydrate antigen 15-3 (CA15-3), and carbohydrate antigen 125 (CA125) were examined in normal controls and pancreatic adenocarcinoma patients. The average serum levels of HE4 (**A**); CA19-9 (**B**); CA15-3 (**C**); and CA125 (**D**) were measured with ELISA kit. A significant increase of HE4, CA19-9, CA15-3, respectively, was observed in pancreatic adenocarcinoma patients’ serum compared to normal control serum (* represents *p* < 0.05; ** represents *p* < 0.01), but not for CA125 (ns represents no significance).

**Figure 4:**
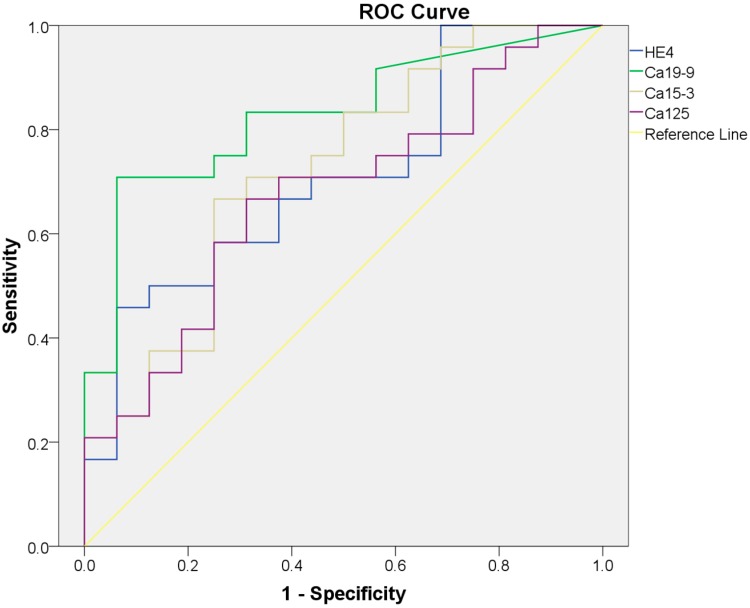
Receiver-operating characteristic (ROC) curves of serum HE4, CA19-9, CA15-3 and CA125. ROC curve of each marker was drawn based on the ELISA results from normal controls and pancreatic adenocarcinoma patients’ serum.

HE4 is a recognized biomarker for ovarian and endometrial cancer. To assess the diagnostic value of HE4 in pancreatic adenocarcinoma, we calculated sensitivity and specificity of HE4 in tumor and controls. ELISA assay was used to detect serum HE4 in 16 control serum samples and 24 tumor samples, and results showed a significant increase of HE4 in tumor serum compared with control (control, 3.58 ± 0.24 ng/mL, tumor, 4.42 ± 0.17 ng/mL; *p* = 0.006) (Figure 3). Serum levels of previously known biomarker for pancreatic cancer CA19-9 and CA15-3 were also significantly higher in cancer patients than normal controls. The receiver-operating characteristic (ROC) curves for HE4 and other biomarkers as well as the cutoff points for achieving the best individual accuracy is shown in Figure 4 and Table 3. Regression analysis showed no significant correlation between HE4 levels and other biomarkers (Table 4), pointing to an independent diagnostic value of HE4 for pancreatic adenocarcinoma patients. Importantly, the combination of HE4, with any of the three markers, led to an increased accuracy compared to either individual marker alone (summarized in Table 5), suggesting the potential clinical usage of HE4 in pancreatic adenocarcinoma.

**Table 3:** The value of Area Under the Curve (AUC), Confidence Interval (CI), cutoff, sensitivity and specificity of four serum markers. The value of AUC, CI, cutoff, sensitivity and specificity for each marker (HE4, CA19-9, CA15-3 and CA125) were calculated, respectively, and shown in the table. CI is at 95% confidence interval (95% CI); Cutoff is the best statistical cutoff value.

Marker	AUC	95% CI	Cutoff	Sensitivity	Specificity
HE4	0.71	0.55–0.87	4.59	45.83%	93.75%
CA19-9	0.83	0.70–0.96	62.85	70.83%	93.75%
CA15-3	0.72	0.56–0.89	18.08	66.67%	75.00%
CA125	0.67	0.51–0.84	4.52	66.67%	68.75%

**Table 4:** Correlations among serum levels of HE4, CA19-9, CA15-3 and CA125 in pancreatic adenocarcinoma patients’ serum. There was no correlation between each two serum markers out of the four markers tested (*p* > 0.05).

Marker	CA199	CA153	CA125
HE4	*r* = 0.095, *p* = 0.785	*r* = −0.365, *p* = 0.079	*r* = 0.123, *p* = 0.568
CA199		*r* = −0.230, *p* = 0.280	*r* = −0.239, *p* = 0.260
CA153			*r* = 0.217, *p* = 0.308

**Table 5:** Summary of sensitivity, specificity and accuracy of each biomarker alone or in combination with HE4. The accuracy of each marker was calculated and shown in the table. The sensitivity, specificity and accuracy of the combination of HE4 with CA19-9, CA15-3 and CA125, respectively, were calculated and listed in the table. An increased sensitivity of combination compared to the two serum marker alone was observed in the three combination groups (HE4/CA19-9, HE4/CA15-3, and HE4/CA125). Each serum protein level was classified as detectable level (+) and undetectable level (−) status, the case numbers of (+) and (−) from cancer patients group and normal group were summarized in table.

Marker	Detectable Status	Patient	Normal	Sensitivity	Specificity	Accuracy
HE4	+	11	1	45.83%	93.75%	65%
	−	13	15			
CA19-9	+	17	1	70.83%	93.75%	80%
	−	7	15			
CA15-3	+	16	4	66.67%	75%	70%
	−	8	12			
CA125	+	16	5	66.67%	68.75%	67.5%
	−	8	11			
HE4/CA19-9	+	20	2	83.33%	87.5%	85%
	−	4	14			
HE4/CA15-3	+	21	4	87.5%	75%	82.5%
	−	3	12			
HE4/CA125	+	19	5	79.17%	68.75%	75%
	−	5	11			

## 3. Discussion

In current study, we demonstrated a significant elevated tissue HE4 mRNA levels by real time PCR as well as serum HE4 protein levels by ELISA in pancreatic adenocarcinoma. HE4 expression in pancreatic adenocarcinoma tissues was also detected. IHC results indicated that 49.1% (27/55) of adenocarcinoma stained with medium to strong intensity, whereas no normal and adjacent non-tumorous tissue was found to be stained with medium to strong intensity. For serum levels of HE4, the cutoff at 4.59 ng/mL produced a sensitivity of 45.83% and specificity of 93.75% (Table 3). These are consistent with previous reports [25,26,27,28] and demonstrate the potential value of HE4 serum levels as a promising biomarker for the diagnosis of pancreatic cancer patients.

Comparison of published data raised a possibility for increasing incidence on pancreatic adenocarcinoma in recent years. Considering its poor prognosis, markers for early screening are the key to improve the prognosis. Biomarkers in various body fluids for pancreatic adenocarcinoma have been investigated. In ductal fluid, high level of S100A8 and A9 predicts a worse prognosis [29]. In pancreatic juice, increased levels of miR-205, miR-210, miR-492, and miR-1247 are associated with poor prognosis for pancreatic adenocarcinoma [30]. However, obtaining juice and ductal fluid from patients represent invasive approaches that cannot be used as a screening tool for early stage patients. Serum biomarkers such as CA19-9, CA-50 and CEA showed some value for diagnosis of pancreatic adenocarcinoma as well as assessment of response to chemotherapy and prognosis [31]. Interestingly, HE4 serum levels appear to be especially higher in early stage of pancreatic adenocarcinoma, and appreciable, but not ideal, levels of sensitivity and specificity were obtained in this study. These findings, together with its advantage of noninvasiveness, warrant further investigation on the efficacy of HE4 as a potential early diagnostic or screening tool for pancreatic cancer patients.

Since normal pancreatic tissues from uncomplicated pancreases are difficult, if not impossible to obtain, we used adjacent tissues as “normal” controls in the determination of HE4 mRNA levels. It should be recognized that adjacent tissues are not perfect controls since inflammatory and other cancer-related responses as well as precancerous changes may occur in such tissues. Thus, we could not exclude possible bias from such comparison. On the other hand, results from the IHC of pancreatic adenocarcinoma indicated diminished low levels of HE4 expression in both normal tissue and adjacent tissues. Moreover, significantly higher levels of HE4 expression were detected in pancreatic adenocarcinoma tissues than both normal tissue and adjacent tissues. This result seemed to validate our conclusion on a significantly increased HE4 mRNA expression in pancreatic adenocarcinoma tissues.

It is noteworthy that increased diagnostic value of HE4 has been achieved through combination with other biomarkers [32,33]. In this study, the serum levels of HE4 and previously investigated markers including CA19-9, CA15-3 all elevated in pancreatic adenocarcinoma. Although there was a trend for increased CA125 in pancreatic adenocarcinoma patients, the difference did not reach a statistical significance. This result is in odd with those from some studies [34]. The relatively small sample size could be one reason. The lack of correlation between serum HE4 levels and CA19-9, CA15-3 or CA125 pointed to an independent diagnostic value of HE4. Indeed, combination of HE4 with CA19-9 lead to a significant increase in the sensitivity (83.33%) compared with HE4 (45.83%) or CA19-9 alone (70.83%), albeit the slightly reduction in specificity. Combination of HE4 with CA15-3 led to a substantially enhanced sensitivity (87.5%) capered to HE4 or CA15-3 alone (45.83%, 66.67%, respectively), although only moderate specificity (75%) and fairly high accuracy (82.5%) were achieved (Table 5). The relatively high sensitivity warrant further studies with larger sample size on the possible value of this combination as a screening tool for the detection of pancreatic adenocarcinoma.

The IHC results showed a negative correlation between HE4 IHC scores and clinical stages (Table 2, *r* = −0.31, *p* = 0.02). A literature search found similar findings on the higher HE4 expression in early stage ovarian as well as endometrioid adenocarcinoma of the uterus [35,36,37], which gives us an inspiration that HE4 may also have a diagnostic value for early stage of pancreatic adenocarcinoma. One explanation for such phenomenon could be related to the HE4’s function. It has been reported that HE4 may play an important role in the development of ovarian and endometrial cancers by promoting cancer cell proliferation and invasion [22,24]. Thus, high levels of HE4 expression may be a perquisite for the fast expansion of cancer lesions in their early stage. Although functional study has not been reported in pancreatic cancer cells, it is highly possible that HE4 may exert similar activities. Regardless the exact function of HE4 in the development of pancreatic adenocarcinoma, its high expression in early stage of cancer appears to underscore its potential value as a marker for the early detection or screening of pancreatic adenocarcinomas.

One major limitation of this study is the small sample size especially for the fresh tissues, which will limit the generalization of our findings. By the same limitation, we could not stratify the patients with available fresh tissue samples by different stages and grades to analyze the HE4 mRNA and serum levels in stage- and grade-specific manner. In addition, the survival analysis could not be performed reliably in a small number of patients.

In conclusion, we present data showing an increased level of HE4 mRNA and protein expression in pancreatic adenocarcinoma tissues as well as elevated HE4 serum levels in patients suffering pancreatic adenocarcinomas. Considering the increased diagnostic value by combination of HE4 with other biomarkers, particularly CA19-9 and CA15-3, and the higher protein level in early stage of cancer, HE4 appears to be a promising marker for pancreatic adenocarcinomas. Further validation experiments in larger population are required to determine the efficacy of ELISA-based HE4 serum assay as a non-invasive tool for the diagnosis and/or screening pancreatic adenocarcinomas.

## 4. Experimental Section

### 4.1. Patients and Tissue Samples

This study was approved by the Oncology Institutional Review Board (IRB) of Memorial Health University Medical Center (IRB #2013.07.04, MHUMC). Forty-six tissue samples, including thirty-two pancreatic adenocarcinoma and fourteen adjacent, non-cancerous tissues were obtained from Cuitis & Elizabeth Anderson Cancer Center of Memorial Health. In addition, 40 serum samples including 24 from pancreatic adenocarcinoma patients and 16 control serum samples from non-cancer patients were also collected. All the tissue and serum samples were stored at −80 °C until use. Human pancreatic cancer and normal tissue microarrays were purchased from US Biomax, Inc. (US Biomax, Inc., Rockville, MD, USA. PA805a). The array contains 58 cases of pancreas adenocarcinoma, 10 cancer adjacent tissues and 10 normal pancreas tissues. Seven cancer cases were removed from analysis because of a lack of complete clinical data.

### 4.2. Quantitative RT-PCR

Total RNA was extracted from frozen pancreatic tissues using RNeasy plus Mini Kit (QIAGEN Inc., Valencia, CA, USA) following the protocol provided by the manufacturer. Two micrograms of total RNA was subjected to reverse transcription to synthesize cDNA using the High Capacity RNA-to-CDNA Kit (Applied Biosystem, Foster City, CA, USA). Real-time PCR was carried out using the USB^®^ VeriQuest™ SYBR^®^ Green qPCR Master Mix (2X) (Affymetrix, Inc., Santa Clara, CA, USA). The sequences of HE4 PCR primers were: forward primer, 5'-ATGAAATGCTGCCGCAATGGC-3'; reverse primer, 5'-GTGGCTGGAGCTCAGAAATTGG-3'.

β-Actin mRNA levels were measures using forward primer, 5'-AAGATCAAGATCATTGCTCCTCCTG-3'; reverse primer, 5'-ATTTGCGGTGGACGATGGAG-3'. HE4 mRNA expression levels were standardized by β-actin mRNA levels as the internal reference. PCR conditions: 95 °C for 10 min, followed by 40 cycles at 95 °C for 15 s and at 60 °C for 1 min using ABI 7900HT Fast Real-Time PCR System (Applied Biosystems, Foster City, CA, USA). β-Actin was used as house-keeping gene in this study. PCR products were resolved and visualized by 1.8% TAE agarose/ethidium bromide gel. Results were standardized to the corresponding internal control gene. Relative mRNA expression folds were calculated. Data were showed as mean ± SD.

### 4.3. Immunohistochemistry

Tissue array slides were deparaffinized with 3 changes of Xylene, each for 5 min. The slides were rehydrated by a sequential treatment with 100% ethanol, 2 changes for 5 min each; 95% ethanol, 2 changes for 5 min each; and 80% ethanol, 2 changes for 5 min each. Endogenous peroxidase activity was quenched with 3% H_2_O_2_ at room temperature for 30 min. After rinsing with distilled water twice, each for 2 min, the slides were incubated in Epitope Retrieval Buffer (IHC-101 IHC kit, Bethyl Laboratories, Inc., Montgomery, TX, USA) at 98 °C for 30 min. After cooling at room temperature for 30 min, Slides were exposed to blocking solution for 30 min. Slides were covered with 1:350 diluted primary anti-HE4 polyclonal antibody (rabbit, biolegend, SIG-3731, Sigma, Ronkonkoma, NY, USA) at room temperature for 2 h. Following extensive washing with PBS, anti-rabbit secondary antibody was applied with 1:400 dilution for 1 h. Color development was performed with DAB Substrate (IHC-101, Bethyl Laboratories, Inc., Montgomery, TX, USA). Reactions were stopped and counterstaining was carried out with Gill’s Hematoxylin Solution (SC-24973, Santa Cruz Biotechnology, Inc., Dallas, TX, USA). The slides were dried and mounted with Organo/Limonene Mount medium (SC-45087, Santa Cruz Biotechnology, Dallas, TX, USA). HE4 expression in each specimens was scored based on the staining intensity (0-negative, 1-weak, 2-moderate, 3-strong) multiplied by the percentage of positive cells 0, 1 (1%–24%), 2 (25%–49%), 3 (50%–74%), and 4 (75%–100%). Tissue samples were classified as negative (score = 0), weak (score = 1–4), medium (score = 5–8), or strong (score = 9–12) according to the final score of individual tissue.

### 4.4. ELISA

HE4 plasma concentrations were determined with the use of Human HE4/WFDC2 Immunoassay (DHE400, R&D Systems, Inc., Minneapolis, MN, USA). Plasma CA19-9 levels were measured using CA19-9 (Human) ELISA Kit (KA0207, Abnova, Walnut, CA, USA), plasma CA15-3 levels were measured using Human CA15-3/Mucin-1 (MUC1) ELISA Kit (RAB0375, Sigma, Ronkonkoma, NY, USA). Measurement procedures were carried out following the manufacturers’ instructions and the values were read at recommended wave lengths of 450 nm.

### 4.5. Statistical Analyses

Statistical analyses were performed with SPSS for Windows version18.0 (SPSS Inc., Chicago, IL, USA). Differences in mRNA levels between groups were analyzed using Student’s *t*-test. The HE4, CA199, CA153, CA124 cut-off values, receiver operating characteristic (ROC) curves, and the areas under the curve (AUC) values were calculated. Cases with marker levels above threshold levels were considered to be positive. When the markers were used in combination the test was considered to be positive if one marker reached the threshold, and negative if both markers were negative. Sensitivity and specificity were calculated for individual markers and their combinations. Kruskal–Wallis nonparametric Correlations, was performed for the immunohistochemistry results. Spearman correlation analysis was used to determine the relationship between immunohistochemistry scores and FIGO stages or pathological grades of pancreatic adenocarcinoma tissues. For all statistical comparisons *p* < 0.05 was considered significant.
